# Broadband, sensitive and spectrally distinctive SnS_2_ nanosheet/PbS colloidal quantum dot hybrid photodetector

**DOI:** 10.1038/lsa.2016.126

**Published:** 2016-07-29

**Authors:** Liang Gao, Chao Chen, Kai Zeng, Cong Ge, Dun Yang, Haisheng Song, Jiang Tang

**Affiliations:** 1 Wuhan National Laboratory for Optoelectronics (WNLO) and School of Optical and Electronic Information, Huazhong University of Science and Technology (HUST), Wuhan 430074, China

**Keywords:** PbS colloidal quantum dot, photodetector, SnS_2_ nanosheet, spectrally distinctive

## Abstract

Photodetectors convert photons into current or voltage outputs and are thus widely used for spectroscopy, imaging and sensing. Traditional photodetectors generally show a consistent-polarity response to incident photons within their broadband responsive spectrum. Here we introduced a new type of photodetector employing SnS_2_ nanosheets sensitized with PbS colloidal quantum dots (CQDs) that are not only sensitive (~10^5^ A W^−1^) and broadband (300–1000 nm) but also spectrally distinctive, that is, show distinctive (positive or negative) photoresponse toward incident photons of different wavelengths. A careful mechanism study revealed illumination-modulated Schottky contacts between SnS_2_ nanosheets and Au electrodes, altering the photoresponse polarity toward incident photons of different wavelengths. Finally, we applied our SnS_2_ nanosheet/PbS CQDs hybrid photodetector to differentiate the color temperature of emission from a series of white light-emitting diodes (LEDs), showcasing the unique application of our novel photodetectors.

## Introduction

Photodetectors, a class of optoelectronic devices that measure optical power by converting the incident light into current or voltage outputs, are widely applied as an essential part of spectroscopy, telecommunication, biological imaging and night vision^1, 2, 3, 4, 5^. Photodetectors can be approximately categorized as broadband or selective. Broadband photodetectors, such as Si and InGaAs photodiode^6, 7^, respond to all incident photons with energy higher than their band gaps. Selective photodetectors, also known as narrowband photodetectors, are designed to detect light at a specific wavelength and are generally applied for biomedical imaging and safety surveillance^8^. It would be desirable to build photodetectors that are both broadband and selective, that is, show photoresponse to a wide spectrum and are still spectrally distinctive. Human eyes are designed to take advantage of three classes of receptor, the short-, medium- and long-wavelength-sensitive cones^9^, to allow perception of a colorful image. A broadband photodetector integrated with a trichroic prism or optical filters, such as a charge coupled device (CCD)^10, 11^, could realize such a function but at high cost and a complicated system configuration. Naturally, it is desirable for broadband and spectrally distinctive (BSD) photodetection to be achieved in a single device, thus significantly simplifying system design and reducing the cost. It is a daunting challenge to realize BSD photodetection in a photodetector with only one absorber material; broadband and spectrally distinctive detection is possible only when a device contains two absorber materials of different band gaps (*E*_g1_ and *E*_g2_) and the two photosensitive materials show opposite photoresponse, that is, one demonstrates positive photoconductivity (PPC) and the other demonstrates negative photoconductivity (NPC).

During recent years, the emergence of graphene and layered metal chalcogenide nanosheets has opened a fundamentally interesting and technologically attractive avenue for photodetection because of their high carrier mobility, convenient material integration and compatibility with complementary metal-oxide semiconductor electronics^12^. The sensitization of these two-dimensional (2D) nanosheets with colloidal quantum dots (CQDs, a 0D material) further broadens the response spectrum and achieves ultrahigh responsivity due to the synergism between the strong absorption of CQDs and the extremely fast circulation of carriers within the 2D nanosheet^13, 14^. This 2D–0D hybrid photodetector, which has already demonstrated outstanding performance, would be ideal for BSD photodetection if an additional spectrally distinctive photoresponse could be engineered. Unfortunately, to the best of our knowledge, there are no reports of BSD photodetection in this system, despite gate-dependent, ambipolar light detection being observed in the literature^14^.

Here we report a SnS_2_ nanosheet/PbS CQD 2D–0D hybrid system that demonstrated sensitive, broadband and spectrally distinctive photoresponse, a combination of merits never reported before. The device is a SnS_2_ nanosheet phototransistor sensitized with infrared-absorbing PbS CQDs. When illuminated by short-wavelength ultraviolet light, both SnS_2_ and PbS are excited and contribute to enhanced conductivity, demonstrating a PPC effect; when illuminated by long-wavelength infrared light, only PbS is excited and the Schottky barrier between the Au electrode and the SnS_2_ nanosheet increases, blocking electron transmission to trigger an NPC effect. In such a way, spectrally distinctive photoresponse was obtained, in addition to broad response from ultraviolet to the near-infrared, ultrahigh sensitivity up to 1 × 10^6^ A W^−1^ and excellent weak light detection with measured noise equivalent power (NEP) of 7.89 × 10^−16^ W Hz^−^^1/2^. A semi-quantitative model is followed to elucidate the working principle of our spectrally distinctive photodetectors. Finally, our BSD photodetectors are applied to measure the color temperature of commercial light-emitting diodes (LEDs) to showcase the applications of these BSD photodetectors that are impossible for regular photodetectors.

## Materials and methods

PbS CQDs were synthesized and isolated according to a modified Hines method^15^. The oleic acid-passivated PbS CQDs were dispersed in toluene at a concentration of 5 mg ml^−1^. Tin disulfide (SnS_2_) single crystals were grown by the chemical vapor transport method using iodine as the transport agent according to previous reports^16, 17^. Various layers of SnS_2_ nanosheets were obtained by a micromechanical cleavage method as with graphene exfoliated from graphite. Numerous SnS_2_ nanosheets were transferred onto a SiO_2_/Si substrate with a 300-nm SiO_2_ dielectric layer. Electrodes of Au (100 nm) with a channel gap of 10 μm were patterned using a designed mask and deposited by thermal evaporation. The PbS CQDs in toluene were deposited onto SnS_2_ nanosheets by spin-coating and subsequent ethanedithiol treatment using a solid-state ligand-exchanging process^18^; an additional spin-coating and treatment cycle was performed. Afterwards, the SnS_2_ nanosheet/PbS CQDs device was baked at 90 °C in air for 10 min. As a control, we found that ethanedithiol has a minimum effect on the well-crystallized SnS_2_ nanosheets.

All device performance characterizations were performed in air in an optically and electrically sealed box to minimize electromagnetic disturbance. Temporal response measurement was conducted using a UV LED (Thorlabs M365L2, Newton, NJ, USA) and an NIR LED (Thorlabs M970L3) modulated by a waveform generator (Agilent 33600A Series, Santa Clara, CA, USA). Dark currents and photocurrents were measured using a semiconductor device analyzer (Agilent B1500A) by averaging the current over time for each voltage step. To measure the photoresponse, we illuminated the devices globally with a beam spot of 3 mm and calculated the responsivity from the incident flux in the active area (~300 μm^2^) of the device. The monochromatic light was modulated by optical grating with a minimum step of 10 nm from a xenon lamp. All photocurrents of the photodetector were recorded using a semiconductor device analyzer (Agilent B1500A). Noise currents were measured using a Stanford Research SR850 lock-in amplifier. Batteries were used to bias the device, and special attention was paid to any possible electromagnetic interferences to minimize the effect of external noise. Through the choice of integration time, the lock-in amplifier reported a noise current in A Hz^−^^1/2^.

For the UPS and Kelvin Probe test, one layer of 5 mg ml^−1^ PbS CQDs was spin-coated onto large-area SnS_2_ nanosheets to form discrete PbS CQDs films. The UPS measurement was performed using a Kratos UPS accessory in a vacuum environment. KP measurement was performed using an ambient Kelvin Probe System (KP Technology, Wick, Caithness, Scotland) in non-contact mode in the dark state or with grazing incidence from a UV or NIR LED (Thorlabs M365L2 or M970L3, Wick, Caithness, Scotland). We measured each condition three times, and 100 points were automatically sampled. For color temperature measurement, the white LEDs (Shen Zhen ChunDaXin) were driven by a waveform generator (Agilent 33600A Series) to generate the same emission intensity measured by a thermal radiator (Newport Oriel Instruments, Stratford, CT, USA). The characteristic spectra of LEDs were confirmed using a visible spectrometer (Zolix Omni-500 nm). Under illumination of different white LEDs with the same power density, the photoelectric signals of our 2D SnS_2_/PbS CQD hybrid photodetectors were recorded by a semiconductor device analyzer (Agilent B1500A).

## Results and discussion

**Figure 1:**
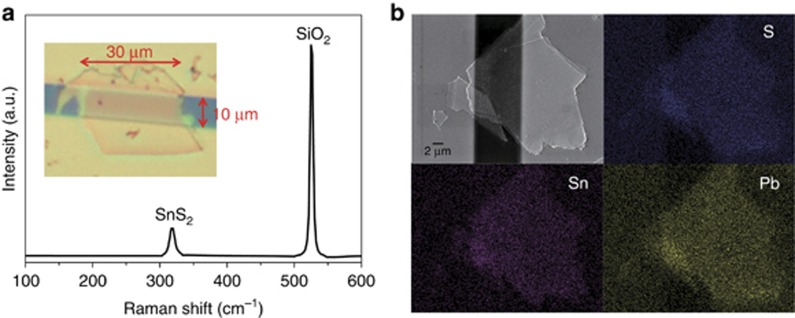
Device structure of our BSD photodetector. (**a**) Normalized Raman spectrum of our mechanically exfoliated SnS_2_ nanosheet. The inset is the optical microscopy image of a SnS_2_ nanosheet with its dimension of ~10 × 30 μm. The blue bar is the gap between yellow Au electrodes. (**b**) SEM image of SnS_2_ nanosheet/PbS CQDs and the corresponding S, Sn and Pb element mapping.

SnS_2_ nanosheets sensitized with PbS CQD were chosen to build the prototypical BSD photodetectors. The SnS_2_ nanosheet serves as the high-mobility carrier transport channel, and PbS CQDs work as the sensitizer, extending the absorption spectrum into the infrared. Hexagonal SnS_2_ has a visible bandgap of 2.2–2.35 eV (refs 19, 20) and a low Fermi level (close to the vacuum) due to its n-type doping originated from sulfur vacancies^21^, thus facilitating the easy construction of Schottky contact with a high work function Au electrode. Few layered SnS_2_ nanosheets from mechanical exfoliation of bulk SnS_2_ single crystals were dispersed onto SiO_2_/Si substrates on which Au electrodes with a spacing of 10 μm were thermally evaporated. Figure 1a shows the Raman spectrum of the as-exfoliated SnS_2_ nanosheets and the optical image of the corresponding phototransistor. Raman characterization showed that the intensity ratio of the SnS_2_ peak to SiO_2_ peak is approximately 1:7, which indicates the thickness of the SnS_2_ nanosheet is ~5 layers^22^. The nanosheet thickness is further confirmed by atomic force microscopy characterization, as shown in Supplementary Fig. S1. The lateral dimension of the SnS_2_ nanosheet is ~30 μm, being covered by the top Au electrodes. We performed the *I*_d_*–V*_g_ characterization (Supplementary Fig. S2), and the calculated electron mobility (*μ*_e_) of our SnS_2_ nanosheet is ~3.33 cm^2^ ( Vs)^−1^, which is comparable to the previous bottom-gate FET result^17, 22^. Because the absorption cutoff of SnS_2_ is ~520 nm, PbS CQDs with excitonic peak at 939 nm (Supplementary Fig. S3) were introduced to widen the absorption spectrum. Bulk PbS has an *E*g of 0.4 eV, and the absorption onset of PbS CQDs synthesized through hot injection could in principle be extended to 3 μm by increasing the physical dimensions^23^, highlighting the potential of PbS CQDs for broadband absorption. Upon isolation, PbS CQDs are capped with a long insulating oleic acid ligand; once spun onto the SnS_2_ nanosheet, a solid state ligand exchange applying ethanedithiol (EDT) to remove the ligands and passivate surface defects was executed^18^. We spin-coated only two layers of PbS CQDs using 5 mg ml^−1^ toluene dispersion to generate a discontinuous CQD film; in such a way, carrier transport in the SnS_2_ nanosheet dominates, whereas parallel transport within the CQD film is negligible. Figure 1b presents the top-view scanning electron microscopy (SEM) image of the finished device. The dark gray rectangle is the gap between the Au electrodes; the shape of the SnS_2_ nanosheet is easily distinguishable from the obvious contrast and clear periphery. Careful examination revealed many white spots on the surface, which were PbS CQDs, as confirmed by the uniform distribution of Pb in the element mapping.

**Figure 2:**
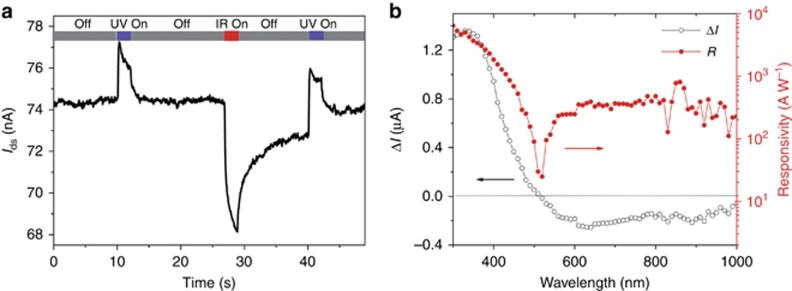
Spectrally distinctive light detection of the SnS_2_ nanosheet/PbS CQD BSD photodetector. (**a**) Drain-source current-time (*I*_ds_–*t*) response under alternate switching of ultraviolet (UV, 365 nm) and near-infrared (NIR, 970 nm) LED illuminations. (**b**) Wavelength-dependent photocurrent and responsivity of our BSD photodetector. The drain-source voltage was 1 V, and no gate bias was applied for these measurements. The light source is a xenon lamp modulated with an optical grating to generate monochromatic light with a minimum step of 10 nm, as shown in Supplementary Fig. S4.

Our SnS_2_ nanosheet/PbS CQD hybrid photodetector showed broad, sensitive and spectrally distinctive photoresponse. As shown in Figure 2a, when illuminated globally by a collimated beam of 3-cm^2^ diameter and biased with drain-source voltage (*V*_ds_) of 1 V and gate voltage (*V*_g_) of 0 V, that is, working as a photoconductive photodetector, the photoresponse polarity of our device was spectrum-dependent. When illuminated by ultraviolet light generated through a 365-nm LED, the current increased, showing positive photoconductivity. In contrast, when illuminated by NIR light generated through a 970-nm LED, the resistance increased, showing negative photoconductivity. Figure 2b demonstrates a more comprehensive picture of the wavelength-dependent photoresponse. Monochromatic light is modulated by the optical grating of a xenon lamp spectrum with a minimum step of 10 nm. The net photocurrent (Δ*I*) (defined as *I*_p_*−I*_d_, where *I*_p_ is the current under illumination and *I*_d_ is the dark current), and the responsivity (defined as *ΔI/P*_in_ in units of A W^−1^, and *P*_in_ is the incident light power), demonstrated a clear transition from negative to positive when the illumination light changed from infrared to ultraviolet. The turning point, which is the wavelength where the photoresponse polarity switched, was 520 nm, as shown as the trough in the red responsivity curve (please also see Supplementary Fig. S5). The observed spectrally distinctive photoresponse is very rare for photodetectors reported in the literature, and the underlying mechanism governing this phenomenon will be discussed in detail later. Our device showed broad photoresponse to the near-infrared, with its cutoff wavelength determined by the PbS CQDs. Furthermore, the responsivity was thousands of A W^−1^, an intrinsic advantage of our 2D/0D hybrid photodetectors. Such high sensitivity is appreciated for applications where sensing extremely weak-intensity light is at a premium.

**Figure 3:**
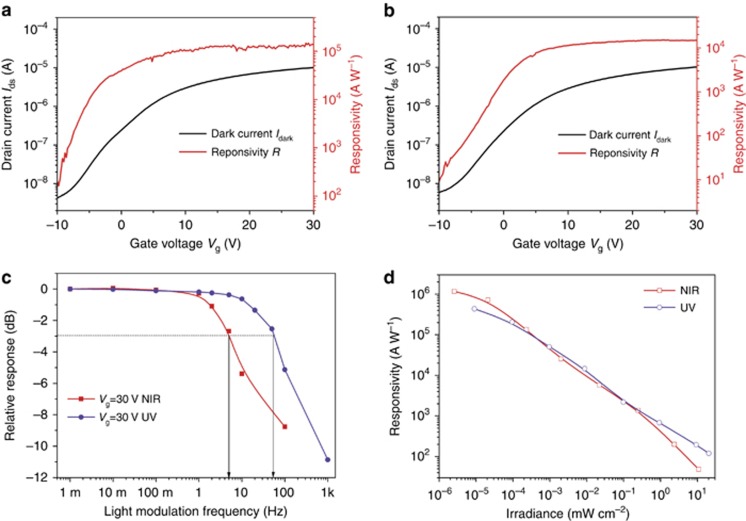
Device characteristics of our SnS_2_ nanosheet/PbS CQD BSD photodetector. Plots of the drain current *I*_ds_ (in dark conditions) and responsivity *R* as a function of the back-gate voltage *V*_g_ under (**a**) NIR (970 nm, 0.23 μW cm^−2^) and (**b**) UV (365 nm, 8.78 μW cm^−2^) illumination. The drain-source voltage is 1 V for these measurements. Please read the black (red) curves corresponding to the black (red) *Y*-axes. (**c**) Normalized NIR and UV response of the hybrid photodetector versus the input signal frequency (both NIR and UV modulation frequency at an intensity of ~1 mW cm^−2^) at *V*_g_=30 V. The −3 dB point is specified with the arrow. (**d**) Light intensity-dependent NIR and UV photoresponse for our hybrid photodetector. Measuring conditions: *V*_ds_=1 V and *V*_g_=30 V. The light intensity varies over 6 orders of magnitude.

We now turn to the optoelectronic figures of merit for the hybrid photodetector under NIR and UV illumination, as shown in Figure 3a and 3b, respectively. Considering the negative photoconductivity observed under NIR illumination, responsivity is calculated as |*I*_d_*−I*_p_|/*P*_in_. At negative gate voltage (*V*_g_), the gating depletes the n-type SnS_2_ nanosheet, decreasing the dark current, operating in OFF mode; by increasing *V*_g_, the SnS_2_ channel falls in the accumulation region, increasing the dark current and operating in the ON state. A similar trend was observed for responsivity. As *V*_g_ swept from −10 to 30 V, the responsivity increased steeply at the beginning and then gradually saturated. The saturating behavior at high *V*_g_ is due to the sufficiently large gate electric field, making the electrons reach saturation concentration^24^. For both UV and NIR illumination, our hybrid photodetector demonstrated high responsivity in excess of 10^5^ A W^−1^, which is much larger than that of PbS CQDs photodetectors (~10^3^ A W^−1^)^8^ or single SnS_2_ nanosheets phototransistors (~10^2^ A W^−1^)^22^, a proven advantage of the 2D/0D device configuration that takes advantage of fast carrier transport in the SnS_2_ channel. After photoexcitation, when holes are trapped inside PbS CQDs and/or the SnS_2_ nanosheet by defects, electrons circulate multiple times through the SnS_2_ transmission channel simultaneously, giving rise to the high gain^13, 14, 25^.

Figure 3c displays the frequency domain response, and the UV response speed is much faster than that of the NIR. The 3-dB frequency, defined as the frequency at which response dropped to 0.707 of the initial value, was 50 Hz for UV response. The 3-dB frequency of the NIR response was only 5 Hz due to the longer lasting defects in PbS CQDs or the carrier transfer process from PbS CQDs to the SnS_2_ nanosheet. We also measured the dynamic range of our hybrid photodetector, and the results are shown in Figure 3d. The responsivity decreased as the light intensity increased, a phenomenon frequently observed in photoconductivity photodetectors due to bimolecular recombination between free holes and electrons under high-power illumination, in addition to the saturation of sensitizing traps in PbS CQDs and/or SnS_2_ nanosheets that contribute to the gain^26, 27^. We derived the shot-noise limited detectivity (D*), a noise that is associated with current fluctuation and could be directly calculated from the dark current, to be >10^14^ Jones for the NIR and >10^13^ Jones for ultraviolet detection. However, this value is heavily overestimated because the measured noise in the dark presented a strong 1/*f* component (Supplementary Fig. S6), which was neglected in the estimation. We thus measured the noise spectrum using a lock-in amplifier and calculated the NEP as 7.89 × 10^−16^ and 7.23 × 10^−15^ W Hz^−1/2^ for NIR and UV illumination, respectively. The detectivity was further estimated as 2.2 × 10^12^ Jones and 2.4 × 10^11^ Jones for NIR and UV illumination, following equation (1), where *A* is the device area in cm^2^ and Δ*f* is the working frequency (1 Hz):


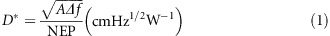


We summarized our device performance along with a few representative results from the literature in Table 1. Clearly, our hybrid photodetector showed outstanding balanced performance, in addition to unprecedented spectral distinction.

**Figure 4:**
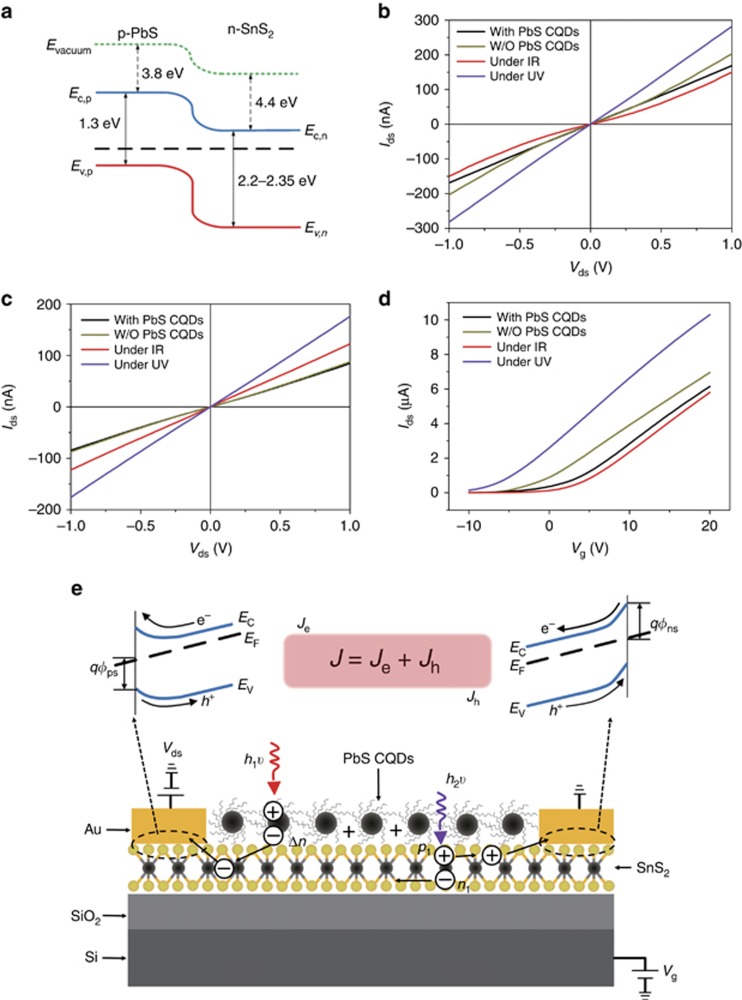
Mechanism study of our BSD photodetector. (**a**) Band diagram between the SnS_2_ nanosheets and EDT-treated PbS CQDs. (**b**) Current–voltage (*I*_ds_–*V*_ds_) curves for devices using Au electrodes and (**c**) *I*_ds_–*V*_ds_ curves for devices using Ti/Au electrodes. The red and dark yellow curves in **b** are nonlinear. The notes in **b**–**d** ’With PbS CQDs,’ ’W/O PbS CQDs,’ ’under IR‘ and ’under UV‘ represent SnS_2_ nanosheets/PbS CQDs device in the dark, SnS_2_ nanosheet-only device in the dark, SnS_2_ nanosheets/PbS CQDs device under NIR illumination and SnS_2_ nanosheets/PbS CQDs device under UV illumination, respectively. UV illumination: 365 nm, 1.025 mW cm^−2^; NIR illumination: 970 nm, 1.048 mW cm^−2^. (**d**) Transfer curves of the device using an Au electrode. The mobilities calculated from the linear zones are all ~10 cm^2^ V^−1^ s^−^^1^. (**e**) Schematic diagram of our BSD photodetector in operation. Large black spheres with slim curves are PbS CQDs. Small black and light-yellow spheres connected with short bold lines are Sn and S atoms in the SnS_2_ nanosheets. Upon illumination, electrons and holes generated and circulated within the SnS_2_ nanosheets and crossed the Schottky barrier with Au contacts. The barrier height for electrons (holes) is denoted by *ϕ*_ns_(*ϕ*_ps_). Under bias (left electrode: positive; right electrode: negative), both electrons and holes encounter one barrier during their circulation.

Having characterized the photodetector performance, we now discuss the working principle in detail. First, the band diagram between the n-SnS_2_ nanosheets and the p-PbS CQD film is shown in Figure 4a. Because our SnS_2_ nanosheets are five layers thick, the conduction band (CB) and valence band (VB) positions are adopted from the bulk values^28, 29^. Naturally, charge transfer could occur at the SnS_2_ nanosheet/PbS CQD interface, permitting photogenerated electron flow from PbS CQDs to SnS_2_ nanosheets and holes from SnS_2_ nanosheets to PbS CQDs. Upon illumination, electrons injected from PbS CQDs or generated within SnS_2_ nanosheets would circulate through the SnS_2_ channel driven by the applied electric field, leading to photoresponse. Second, non-Ohmic contact was observed between Au electrodes and pristine SnS_2_ nanosheets (Figure 4b, dark yellow curve); once EDT-treated PbS CQDs were deposited onto SnS_2_ nanosheets, a linear current–voltage (*I*–*V*) curve was obtained (Figure 4b, black line) in the dark, suggesting the formation of Ohmic contact. The Fermi level of the n-SnS_2_ nanosheets was established as −4.62 eV, derived by subtracting the intercept at a binding energy of 16.58 eV with the ultraviolet photon energy (UPS, He I excitation, 21.2 eV, Supplementary Fig. S7), thus forming a Schottky contact with Au whose work function is −5.1 eV. After coating SnS_2_ nanosheets with p-type PbS CQDs, holes, which are the majority carriers, diffused into the n-type SnS_2_ nanosheets under the carrier concentration gradient, acting like p-type doping, and shifted the Fermi level of SnS_2_ close to the valence band. UPS measurement confirmed this conjecture. The Fermi level of the SnS_2_ nanosheet decreased to −4.79 eV after PbS CQDs coating, accounting for the formation of Ohmic contact with Au electrodes. Upon NIR illumination, only PbS CQDs are absorbed and photogenerated electrons flow into SnS_2_ nanosheets, reversing the p-type doping effect in the dark and shifting the Fermi level of SnS_2_ nanosheets upwards. Consequently, the contact between SnS_2_ nanosheets and Au electrodes is again Schottky, as evidenced by the tortuous current-to-voltage curve (Figure 4b, red curve), accounting for the observed negative photoconductivity. Upon UV illumination, SnS_2_ nanosheets are strong absorbing, and the carrier density increases sharply in the transmission SnS_2_ channel, which could override the negative impact by PbS CQDs, leading to the observed positive photoconductivity (Figure 4b, purple line).

We provided three pieces of experimental evidence to support our analysis. First, because UPS is not capable of measuring the Fermi level under illumination in our facility, we used Kelvin probe measurements. By averaging 100 points, the measured Fermi levels of the SnS_2_ nanosheet/PbS CQD composite in the dark, illuminated by a 970-nm LED (1.048 mW cm^−2^) or a 365-nm LED (1.025 mW cm^−2^) were −4.74, −4.50 and −4.79 eV, respectively. NIR illumination significantly upshifted the Fermi level of the SnS_2_ nanosheets, whereas UV illumination had a negligible effect. Second, we fabricated identical devices using 10 nm Ti/100 nm Au instead of pure Au as the contacts. Because the work function of Ti (4.3 eV) is lower than that of Au, Ohmic contacts were obtained between the SnS_2_ nanosheet and the Ti/Au electrodes. Under this scenario, linear *I*–*V* curves and positive photoconductivity were consistently observed, whether under NIR or UV illumination (Figure 4c, Supplementary Fig. S8). Third, the transfer curves shown in Figure 4d indicate that PbS CQDs have a small effect on the carrier mobility of SnS_2_ nanosheets, ruling out the possibility that charge scattering by the positive photo-charged PbS CQDs has a leading role^14^ for the observed negative photoconductivity. Collectively, we concluded that the Schottky barrier between the Au electrode and the SnS_2_ transmission channel, with its height modulated by external illumination, accounted for the peculiar spectrally distinctive photoresponse observed in our hybrid SnS_2_ nanosheet/PbS CQD photodetector.

We would like to present a physical model to describe the observed spectrally distinctive photoresponse. Considering charge circulation mainly within the SnS_2_ nanosheets (the PbS CQDs film is discontinuous; although there might be transport pathways, the carrier mobility is much worse than in SnS_2_ nanosheets), we define the contact barrier between the SnS_2_ nanosheet and the Au electrode for electrons and holes as *ϕ*_ns_ and *ϕ*_ps_, respectively. During operation, the barrier height is not affected by the external bias, and charge flow through a Schottky barrier is described by the thermal emission model^30, 31, 32, 33^. Consequently, the current density in the dark (*J*_d_) is the sum of contributions from electrons (*J*_e_) and holes (*J*_h_) in the dark


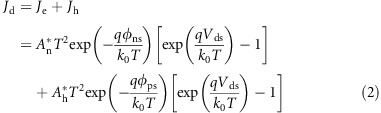


where *A*_n_^*^, *A*_h_^*^, *T* and *k*_0_ are the effective electron Richard constant, effective hole Richard constant, temperature and Boltzmann constant, respectively. Upon illumination, PbS CQDs and/or SnS_2_ nanosheets absorb incident photons and generate carriers, which further transfer between each other. Here we denote photogenerated electrons and holes, under equilibrium, in SnS_2_ nanosheets by *n*_1_ and *p*_1_, respectively, and the electrons (holes) injected from PbS CQDs (SnS_2_ nanosheets) to SnS_2_ nanosheets (PbS CQDs) by Δ*n* (Δ*p*). We also denote the original free electrons and holes concentrations in the SnS_2_ nanosheets by *n* and *p*, respectively. By correlating the carrier density with the Fermi energy and further with the barrier height, we finally formulated the following equation (please refer to Supplementary Fig. S9 for detail):


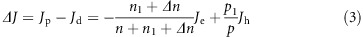


where Δ*J* is the net photocurrent density, and *J*_p_ is the current density under illumination. Δ*J* is dependent on multiple variables, including *n*, *n*_1_ and Δ*n*, which depend on the incident light intensity, PbS CQDs and SnS_2_ film thickness and doping density, and the channel geometry. Nonetheless, the observed wavelength-dependent photoresponse is easily explainable by equation (3). Upon NIR illumination, only PbS CQDs absorb incident photons so *n*_1_=*p*_1_=0, and equation (3) is simplified as:


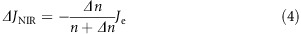


Δ*J*_NIR_ should be negative, explaining the observed negative photoconductivity upon infrared illumination. This conclusion remains valid for illumination with a wavelength below the absorption onset of SnS_2_ nanosheets. Upon UV illumination, both SnS_2_ nanosheets and PbS CQDs are excited. Holes remain in PbS CQDs, and electrons are injected into the SnS_2_ nanosheets (Δ*n*). The photo-induced positively charged PbS CQDs repel hole injection from SnS_2_ nanosheets, leading to negligible Δ*p* (ref. 34). In addition, because *J*_e_ and *J*_h_ are comparable due to the identical barrier height, the Δ*J*_UV_ value mainly depends on the *p*_1_/*p* ratio. *p* should also be small because the SnS_2_ nanosheet is an n-type semiconductor with a wide bandgap. When incident ultraviolet photons are heavy, *p*_1_>*p* and positive photoconductivity is obtained; when ultraviolet illumination is weak, *p*_1_ is comparable or even smaller than *p*, and negative photoconductivity is expected. This assumption is confirmed by our experimental results (Supplementary Fig. S5b). Under the illumination of 530 nm LED, the photoresponse turns from negative to positive with increasing light power density, verifying that increasing of *p*_1_ turns the photoresponse (Δ*J*) positive.

**Figure 5:**
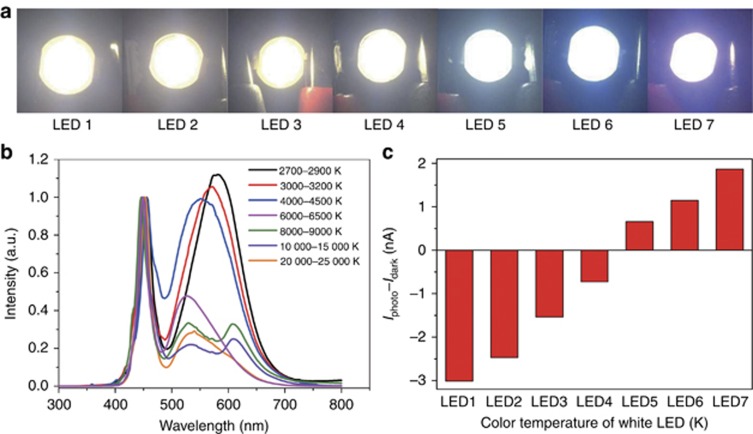
Measurement of the color temperature of white LEDs using our BSD photodetector. (**a**) Photos of seven LEDs with their color temperatures increasing gradually from left to right. The output power density of all LEDs is 0.5 mW cm^−2^. (**b**) Normalized emission spectra of the seven LEDs. (**c**) Net photocurrent of our SnS_2_ nanosheet/PbS CQD hybrid photodetectors to the seven LEDs with the same power density of ~0.5 mW cm^−2^.

We further demonstrated a unique application of our hybrid photodetector for color temperature differentiation of a series of white LEDs. The color temperature, usually measured in K, reflects the spectral resemblance of a light source to a black body radiator. Low color temperature implies warmer (more yellow/red component) light, whereas high color temperature implies cooler (more blue/violet component) light. Figure 5a presents the digital photos of seven LEDs emitting white light with the same intensities, as calibrated by a balometer. The color temperature of these LEDs, however, showed significant increases from left to right, as evidenced by the blue halo in LED6 and violet halo in LED7. This difference is more apparent in the normalized wavelength-dependent emission in Figure 5b: the red emission components in the 500–700 nm range gradually decreased when the color temperature of these LEDs increased. Conventional Si photodetectors hardly distinguish these color temperature differences because they show indiscriminate response to all incident photons and hence generate similar outputs. For our BSD photodetector, however, the photoresponse is significantly different because of its spectrally distinctive characteristics. For LED1, with the lowest color temperature and hence strongest red photons, our photodetector showed strong negative photoresponse because negative photoconductivity from red photons overrode the positive photoconductivity from blue photons. In contrast, the photodetector showed large positive photoresponse toward LED7 because of its high color temperature and large portion of blue photons. Although the precise determination of color temperature is beyond the scope of this study because careful calibration and known emissivity values are required, our preliminary results showcased the novel applications of our hybrid photodetector brought about by the unique spectrally distinctive characteristics.

Finally, we discuss the advances of our work in brief. Sensitization of 2D materials (graphene, MoS_2_ and SnS_2_) with another absorbing material combines the fast transport and easy integration of 2D materials with strong and tunable absorption in the sensitizer, thus providing a versatile hybrid material platform for ultrasensitive and broadband photodetection. Engineering the contacts between 2D materials and electrodes via Schottky barrier modulation further introduced appealing functionality, spectral distinction, into this already promising hybrid system. Experimental results and theoretical analysis revealed that the band gap of 2D materials largely dictated the transition point, the wavelength at which positive and negative photoresponse delineates. The hybrid strategy demonstrated here is easily extendable to different material combinations for broadband, sensitive and spectrally distinctive light detection. Ultimately, we could customize the channel material and the sensitizer and thus produce BSD photodetectors with pre-defined response spectrum and turning point, which might find competitive applications where knowledge of the spectrum range of the incident photons, in addition to their intensities, is required.

## Conclusions

In conclusion, we introduced a new broadband, sensitive and spectrally distinctive photodetector based on SnS_2_ nanosheet/PbS CQD hybrid using Au as the Schottky contact. The device demonstrated ultrahigh sensitivity (>10^6^ A W^−1^) and decent detectivity (>10^12^ Jones), and more importantly, it showed negative photoresponse toward low-energy photons and positive photoresponse toward high-energy photons. The underlying mechanism was carefully studied and ascribed to the illumination-modulated barrier height between the Au electrode and the SnS_2_ nanosheet. In addition, by virtue of its spectral distinction, our BSD photodetector was successfully applied to differentiate the color temperatures of a series of white LEDs. Overall, the strategy described here provided new data regarding the design of a new type of photodetector that is not only sensitive and broadband but can also distinguish the wavelength range of incident photons, thus enabling novel applications impossible for traditional photodetectors.

## Figures and Tables

**Table 1:** Device performance comparison between our device and similar photodetectors employing single components or other hybrids

	Reference	Electric field (V μm^−1^)	Dark current (A)	Spectral coverage (nm)	Responsivity (A W^−1^)	Decay time (ms)
SnS_2_	^22^	2	3.2 × 10^−5^ (*V*_g_=10 V)	<600	1 × 10^2^	~150
PbS QDs	^8^	20	1 × 10^−^^8^ (*V*_g_=0)	400–1500	2.5 × 10^3^	20–30
MoS_2_/PbS	^14^	0.33	2.6 × 10^−^^7^(*V*_g_=−100 V)	400–1500	6 × 10^5^	300-400
G/PbS	^13^	0.05	5 × 10^−3^ (*V*_g_=0)	400–1500	1 × 10^7^	1700
SnS_2_/PbS	This work	0.1	4.5 × 10^−9^(*V*_g_=−10 V)	400–1000	1 × 10^6^ (NIR) 3 × 10^5^ (UV)	160–420 ~20
