# Supplementary material for: Broadband, sensitive and spectrally distinctive SnS2 nanosheet/PbS colloidal quantum dot hybrid photodetector
Source: Light Sci Appl. 2016 Jul 29;5(7):e16126–. doi: 10.1038/lsa.2016.126 (PMC6059941; doi:10.1038/lsa.2016.126)
Supplement: Supplementary Information [file lsa2016126x1.docx]

**Supplementary Information for**

**Broadband, sensitive and spectrally distinctive SnS_2_ nanosheet/PbS colloidal quantum dot hybrid photodetector**

Liang Gao, Chao Chen, Kai Zeng, Cong Ge, Dun Yang, Haisheng Song, Jiang Tang*

Wuhan National Laboratory for Optoelectronics (WNLO) and School of Optical and Electronic Information, Huazhong University of Science and Technology (HUST), Wuhan, 430074, China.

* Corresponding author: [jtang@mail.hust.edu.cn](mailto:jtang@mail.hust.edu.cn)


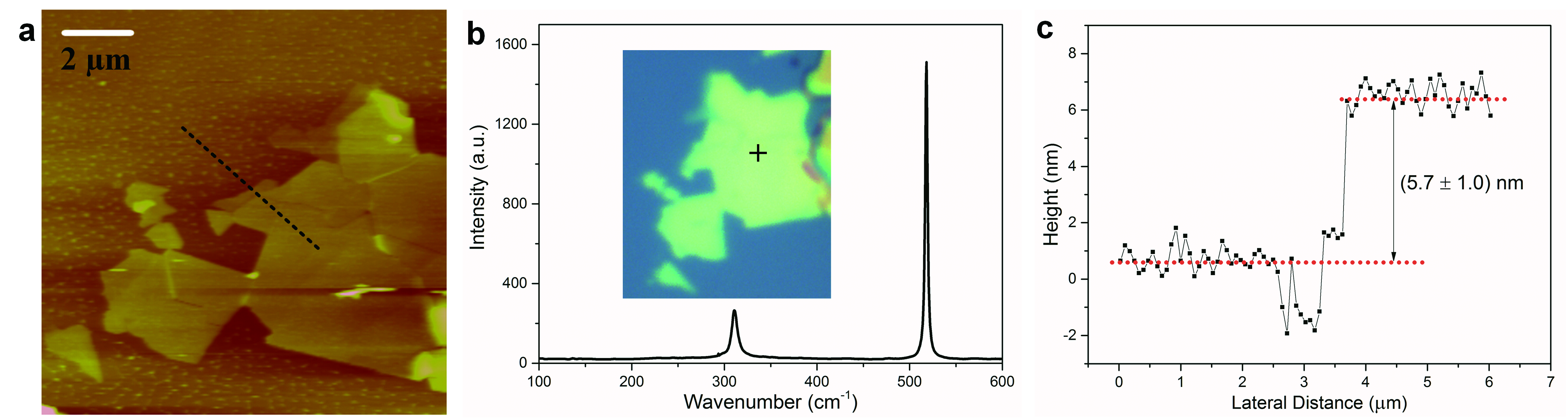


**Figure S1.** Thickness confirmation of SnS_2_ nanosheet. (a) Atomic force microscopy (AFM) image of a SnS_2_ flake. (b) Typical Raman spectrum of the SnS_2_ nanosheet in panel a. The intensity ratio of SnS_2_ peak (512 nm) to Si peak (310 nm) is about 1:7. The inset is the optical image. (c) The height profile along with the black dashed line in panel a; film thickness is 5.7 nm corresponding to SnS_2_ nanosheet of 5 layers.


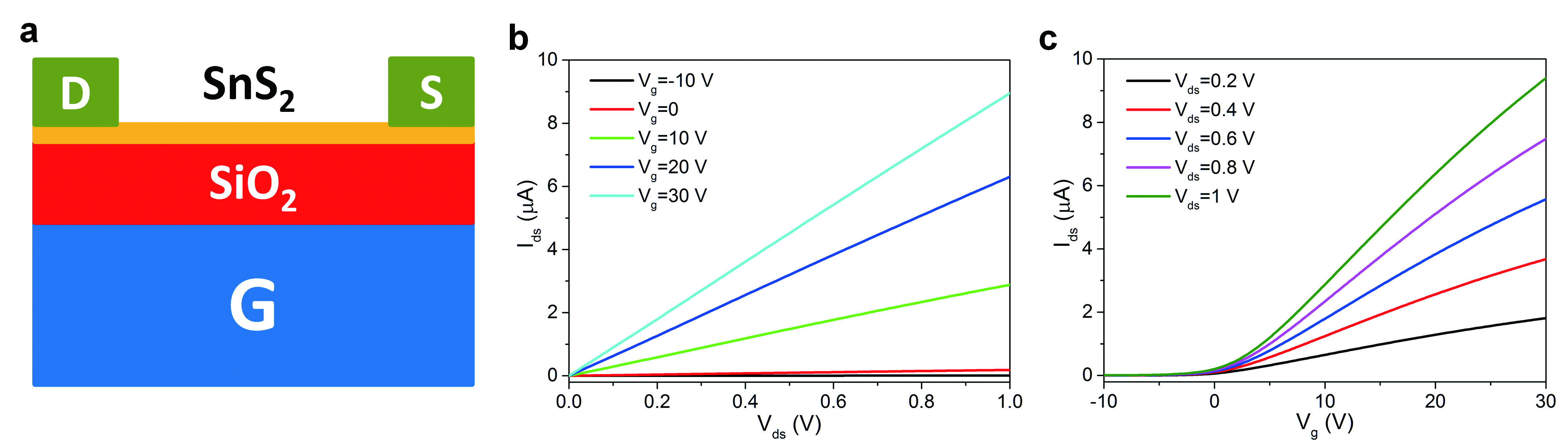


**Figure S2.** Single SnS_2_ nanosheet field effect transistor (FET). (a) Structure diagram of single SnS_2_ nanosheet device. (b) Output characteristic at different gate voltages. (c) Transfer characteristic at different drain voltages.

The drain and source are Au electrodes. The ﬁeld eﬀect mobility of this single SnS_2_ device can be estimated based on the equation

$$\mu=\frac{L}{W\times(\varepsilon_{0}\varepsilon_{r}/d)\times V_{ds}}\times\frac{{dI}_{ds}}{dV_{g}}$$

where the channel length *L* is 10 $\mu m$, the channel width *W* is 30 $\mu m$, $\varepsilon_{0}$ is $8.854\times{10}^{-12} Fm^{-1}$, $\varepsilon_{r}$ for SiO_2_ is 3.9, and the thickness of SiO_2_ *d* is 300 nm. When the *V_ds_* is 1 V, the calculated mobility of our device is about 10 $\mathrm{cm}^{2}V^{-1}s^{-1}$, which is slightly higher than previous results from the bottom-gate FET reported in other reference[^1^](#_ENREF_1)^,^[^2^](#_ENREF_2). The high-mobility SnS_2_ nanosheet can provide a fast channel for photo-generated electron from PbS CQDs.

**
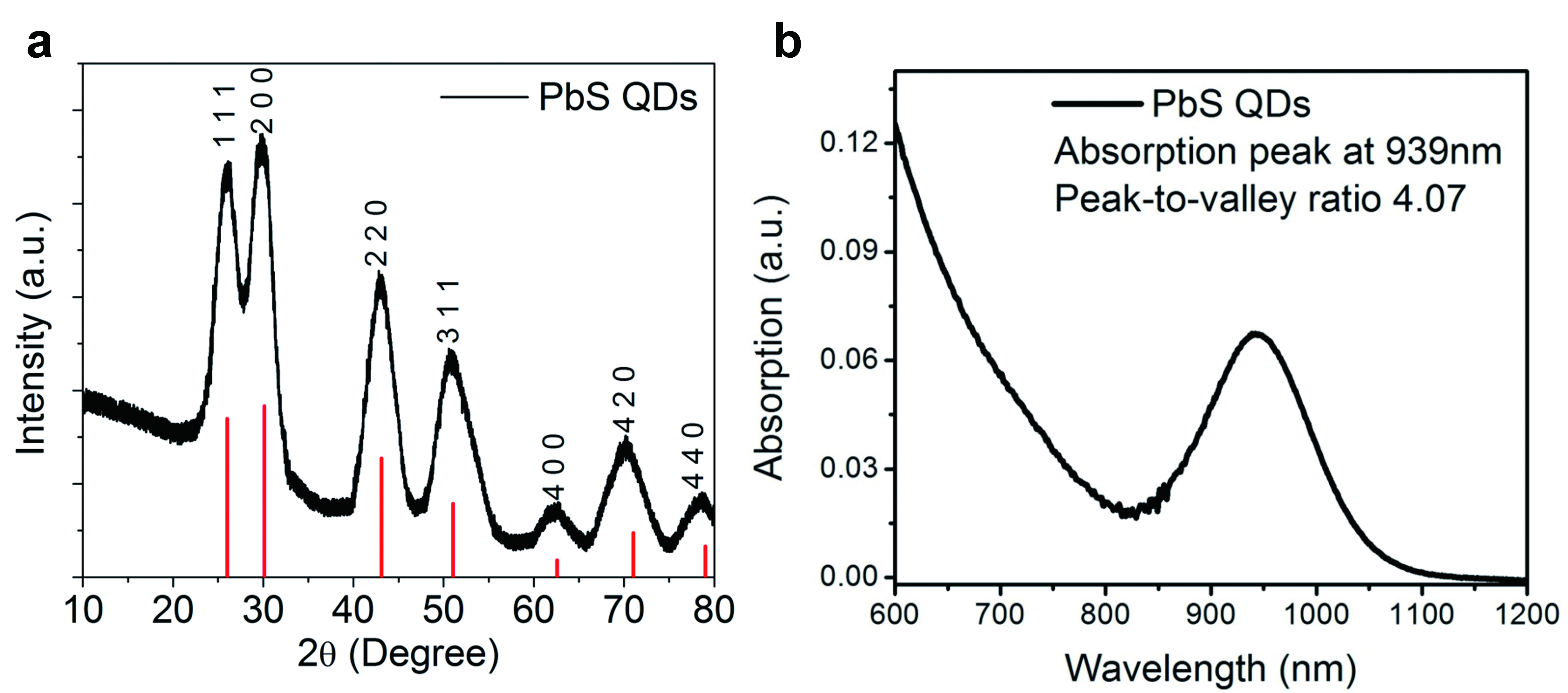
**

**Figure S3.** (a) X-ray diffraction (XRD) pattern and (b) Optical absorption spectrum of PbS CQDs.

The hexagonal SnS_2_ has a visible-light band gap of 2.2-2.35 eV.[^3^](#_ENREF_3)^,^[^4^](#_ENREF_4) Single SnS_2_ nanosheet device cannot respond to infrared light. However PbS CQDs are very sensitive to near-infrared (NIR) light[^5^](#_ENREF_5), we thus introduced PbS CQDs with excitonic peak at 939 nm to broaden the response spectrum.


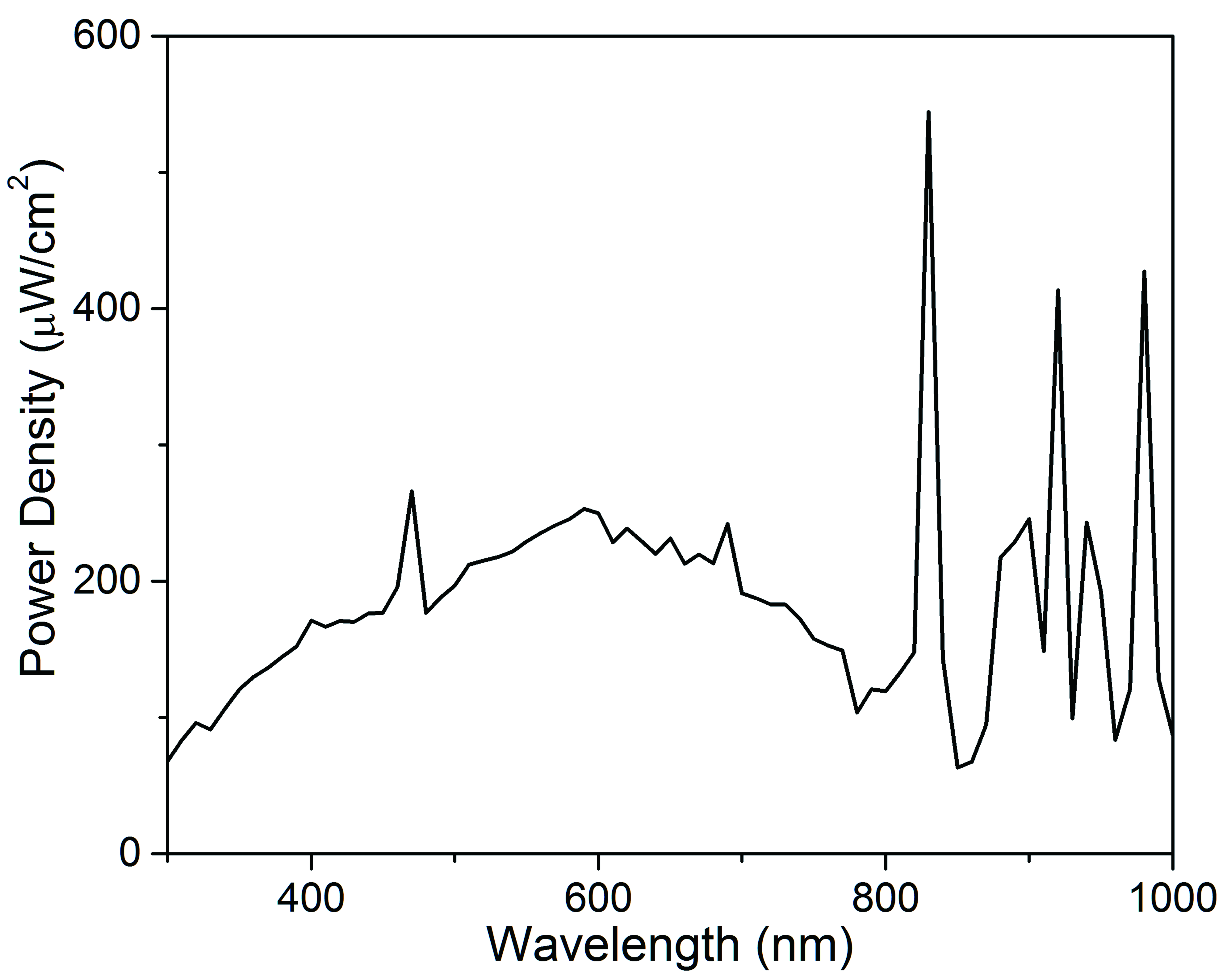


**Figure S4.** Spectrum of the Xenon lamp. It is modulated by optical grating to generate monochromatic light with a minimum step of 10 nm.


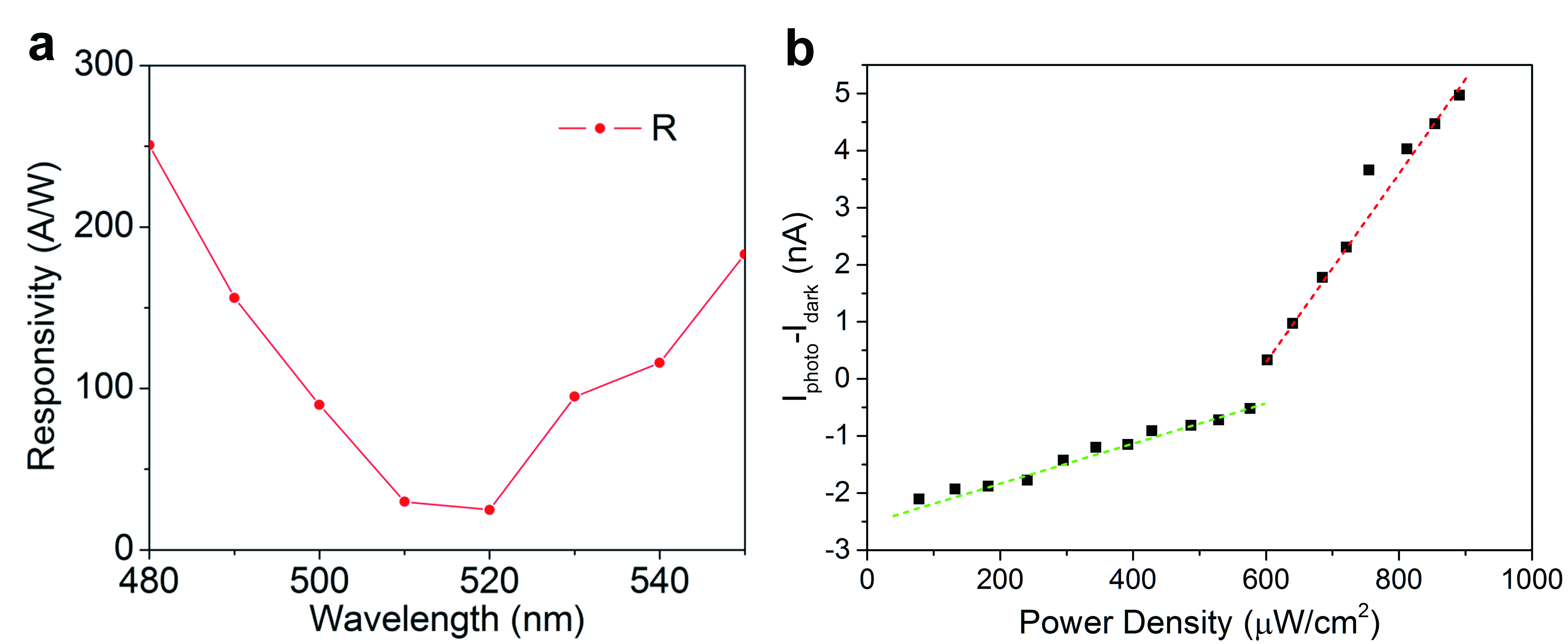


**Figure S5.** (a) The magnified part of responsivity to wavelength shows the turning point is about 520 nm. (b) The photoresponse under 530 nm LED with different power density. Polarity changed when the power density surpass a threshold of ~ 600 μW/cm^2^ for this specific device.


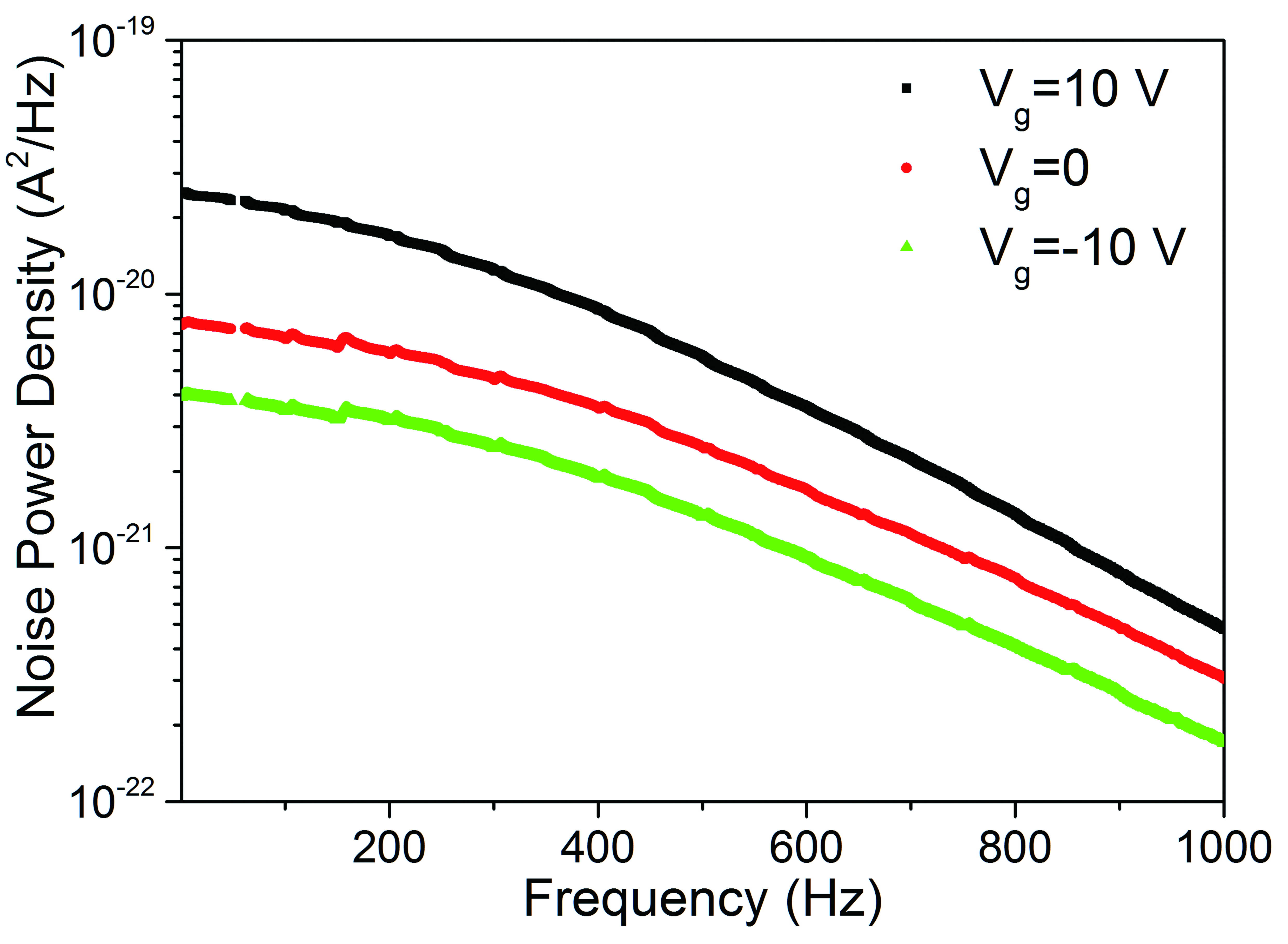


**Figure S6.** Noise power density of a typical SnS_2_/PbS hybrid measured at different backgate voltages for a constant drain-source bias of 1 V.

To analyse the noise-equivalent-power (*NEP*) and detectivity (*D^*^*) of the hybrid detectors, we measured noise for different back-gate voltages at drain-source voltage V_ds_=1 V. The noise spectral density is plotted for back-gate voltage V_g_=10 V, 0, -10 V. With the measured NIR and UV responsivity of 1.1 x 10^5^ A/W and 1.2 x 10^4^ A/W at a modulation frequency of 1 Hz, this hybrid yields NIR and UV *NEP* of 7.89 x 10^-16^ and 7.23 x 10^-15^ W/(Hz)^1/2^. The active area of the hybrid is 300 ${\mu m}^{2}$, the NIR and UV *D^*^* are 2.2 x 10^12^ Jones and 2.4 x 10^11^ Jones at 1 Hz respectively.





**Figure S7.** The Fermi level evolution of SnS_2_ in different condition. (a) Ultraviolet photoelectron spectrum (UPS) of SnS_2_ nanosheet. The sample is a large-area SnS_2_ nanosheet pasted on SiO_2_/Si substrate. He I excitation is 21.2 eV. Linear fitting (red dashed line) is for the Fermi level of SnS_2_. (b) The UPS of PbS/SnS_2_ hybrid. In the bottom sheet, the Fermi levels are measured by Kelvin Probe. D is the difference value between samples and probe. FL is the Fermi level.

For UPS and Kelvin Probe test, one layer of 5 mg/mL PbS CQDs was spin coated on large-area SnS_2_ nanosheets to form discrete PbS CQDs film. The Fermi level of SnS_2_ nanosheet was established as -4.62 eV, derived by subtracting the intercept at binding energy of 16.58 eV with the ultraviolet photon energy (He I excitation, 21.2 eV). After coating one layer of PbS CQDs, the Fermi level of SnS_2_ nanosheet shifted down to -4.79 eV. These two results were consistent with the Kelvin Probe results of -4.60 eV and -4.74 eV. And the Fermi level of thick PbS CQDs film was measured about -4.95 eV by Kelvin Probe. When the PbS CQDs/SnS_2_ nanosheet nanocomposite was parallelly illuminated by 970 nm LED (1.048 mW/cm^2^) or 365 nm LED (1.125 mW/cm^2^), the Fermi levels of SnS_2_ were measured about -4.50 eV and -4.79 eV.


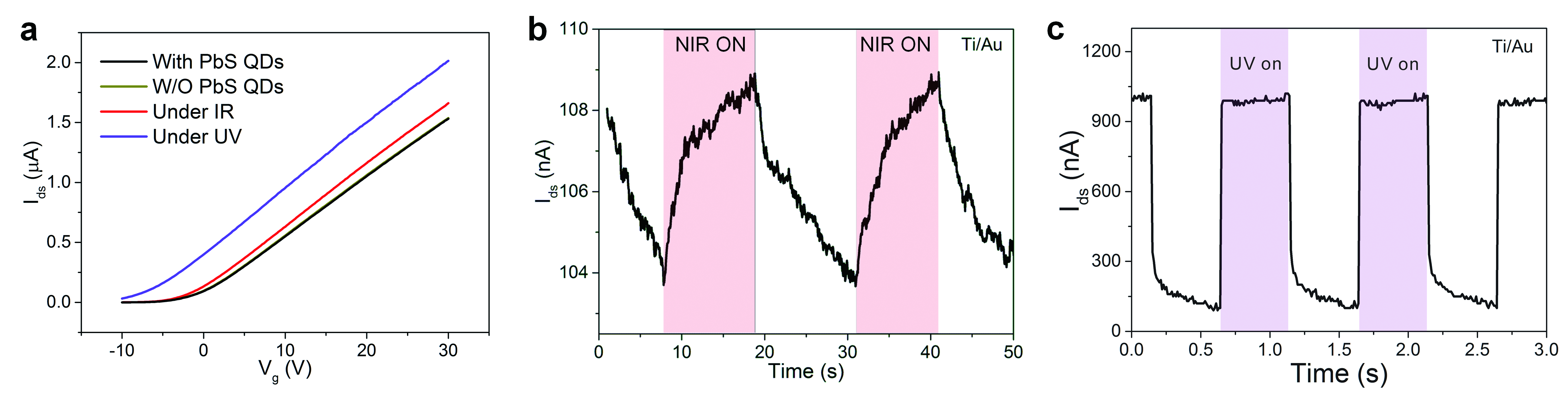


**Figure S8.** Performance of PbS CQDs/SnS_2_ nanosheet device with Ti/Au (10 nm/100 nm) electrodes. (a) Transfer curves of single SnS_2_ device and PbS CQDs/SnS_2_ nanosheet device under 365 nm (1.125 mW/cm^2^) and 970 nm (1.048 mW/cm^2^) LED. (b) Photoswitching behavior of PbS CQDs/SnS_2_ nanosheet device under 970 nm LED (1.048 mW/cm^2^). V_ds_ is 1 V. The cycle time is 10 s. (c) Photoswitching behavior of another PbS CQDs/SnS_2_ nanosheet device under 365 nm LED (1.125 mW/cm^2^). V_ds_ is 1 V. The cycle time is 1 s.

The contact between SnS_2_ nanosheet and Ti/Au (10 nm/100 nm) electrode is always ohmic in any conditions. Under UV and NIR illumination, a normal positive photoconductivity (PPC) effect has been observed.

**Theoretical model and calculation.**

The width of the SnS_2_ channel is *W*, the thickness is *d*, and the length is *L*. The intrinsic electrons density of SnS_2_ is *n*, and the holes density is *p*. The current is calculated through the thermionic emission theory.[^6^](#_ENREF_6) In dark condition, the current density contributed by electrons (*J_e_*) is

 (1)

where $A_{n}^{*}$ is the effective electron Richard constant, and is the electron barrier height between Au electrode and SnS_2_ nanosheet. The current density contributed by holes (*J_h_*) is

 (2)

where $A_{h}^{*}$ is the effective hole Richard constant, and is the hole barrier height between Au electrode and SnS_2_ nanosheet. The total dark current (*J_d_*) is

$J_{d}=J_{e}+J_{h}$ (3)

The barrier height is decided by the quasi Fermi level[^6^](#_ENREF_6), which is related to the carrier density:

$n=N_{c}\exp(-\frac{E_{c}-E_{Fn}}{k_{0}T})$ (4)

$p=N_{v}\exp(-\frac{E_{Fp}-E_{v}}{k_{0}T})$ (5)

These two equations can be evolved into

$E_{c}-E_{Fn}=k_{0}T\ln(\frac{N_{c}}{n})$ (6)

$E_{Fp}-E_{v}=k_{0}T\ln(\frac{N_{v}}{p})$ (7)

Under illumination, electron injection from PbS CQD to SnS_2_ nanosheet is *Δn*, and the reverse holes injection from SnS_2_ nanosheet to PbS CQD is infinitesimal *Δp* due to the repulsive force in these positively photo-charged PbS CQDs[^7^](#_ENREF_7). We also term the photo-generated electrons density in SnS_2_ is *n_1_*, and the holes density is *p_1_*.

$n+n_{1}+\Delta n=N_{c}\exp(-\frac{E_{c}-E_{Fn}^{'}}{k_{0}T})$ (8)

$p+p_{1}-\Delta p=N_{v}\exp(-\frac{E_{Fp}^{'}-E_{v}}{k_{0}T})$ (9)

Since *Δp* is small and can be ignored, these two equations can be evolved to

$E_{c}-E_{Fn}^{'}=k_{0}T\ln(\frac{N_{c}}{n+n_{1}+\Delta n})$ (10)

$E_{Fp}^{'}-E_{v}=k_{0}T\ln(\frac{N_{v}}{p+p_{1}})$ (11)

The electron barrier height variation is

 (12)

 (13)

The current density contributed by photo-generated electrons (*J^’^_e_*) is

 (14)

The current density contributed by photo-generated holes (*J^’^_h_*) is

 (15)

The photocurrent density (*J_p_*) is

$J_{p}=J_{e}^{'}+J_{h}^{'}$ (16)

The photoresponse current density (*ΔJ*)

$\Delta J=J_{p}-J_{d}=-\frac{n_{1}+\Delta n}{n+n_{1}+\Delta n}J_{e}+\frac{p_{1}}{p}J_{h}$ (17)

1 Huang, Y. *et al.* Tin Disulfide An Emerging Layered Metal Dichalcogenide Semiconductor: Materials Properties and Device Characteristics. *ACS Nano* **8,** 10743-10755 (2014).

2 Song, H. *et al.* High-performance top-gated monolayer SnS_2_ field effect transistors and their integrated logic circuits. *Nanoscale* **5,** 9666-9670 (2013).

3 Zhang, Y. C., Du, Z. N., Li, K. W., Zhang, M. & Dionysiou, D. D. High-performance visible-light-driven SnS_2_/SnO_2_ nanocomposite photocatalyst prepared via in situ hydrothermal oxidation of SnS_2_ nanoparticles. *ACS Appl. Mater. Interfaces* **3,** 1528-1537 (2011).

4 Sun, Y. *et al.* Freestanding Tin Disulfide Single-Layers Realizing Efficient Visible-Light Water Splitting. *Angew.* *Chem. Inter. Ed.* **51,** 8727-8731 (2012).

5 McDonald, S. A. *et al.* Solution-processed PbS quantum dot infrared photodetectors and photovoltaics. *Nat. Mater.* **4,** 138-142 (2005).

6 Sze, S. M. & Ng, K. K. *Physics of semiconductor devices*. ***712,*** (John Wiley & Sons, 2006).

7 Scheer, R. & Schock, H.-W. *Chalcogenide Photovoltaics: Physics, Technologies, and Thin Film Devices*. **33,** (John Wiley & Sons, 2011).
